# Supplementary material for: Distinct Clinical Phenotypes in KIF1A-Associated Neurological Disorders Result from Different Amino Acid Substitutions at the Same Residue in KIF1A
Source: Biomolecules. 2025 May 2;15(5):656. doi: 10.3390/biom15050656 (PMC12109325; doi:10.3390/biom15050656)
Supplement: Supplementary file 1 [file biomolecules-15-00656-s001.zip › biomolecules-3543000-supplementary.pdf]

```
kif21b      ----------MAGQGDCCVKVAVRIRPQLSKEKIEGCHICTSVTPGE--------PQVLL   42
kif1a       -------------MAGASVKVAVRVRPFNSREMSRDSKCIIQMSGST---TTIVNPKQPK   44
kif5b       ----------MADLAECNIKVMCRFRPLNESEVNRGDKYIAK----------FQGEDTVV   40
kif3a       ---MP-INKSEKPESCDNVKVVVRCRPLNEREKSMCYKQAVSVDEMRGTITVHKTDS-SN   55
kif11       MASQPNSSAKKKEEKGKNIQVVVRCRPFNLAERKASAHSIVECDPVRKEVSVRTGGLADK   60
                              ::*  * **    *     :   .

kif21b      GKDKAFTYDFVFDLDT--------WQEQIYSTCVSKLIEGCFEGYNATVLAYGQTGAGKT   94
kif1a       ETPKSFSFDYSYWSHTSPEDINYASQKQVYRDIGEEMLQHAFEGYNVCIFAYGQTGAGKS   104
kif5b       IASKPYAFDRVFQSST--------SQEQVYNDCAKKIVKDVLEGYNGTIFAYGQTSSGKT   92
kif3a       EPPKTFTFDTVFGPES--------KQLDVYNLTARPIIDSVLEGYNGTIFAYGQTGTGKT   107
kif11       SSRKTYTFDMVFGAST--------KQIDVYRSVVCPILDEVIMGYNCTIFAYGQTGTGKT   112
               * :::*  :   :         * ::*      ::.  : ***  ::*****.:**:

kif21b      YTMGTGFDMA-----TSEEEQGIIPRAIAHLFGGIAERKRRAQEQGVAGPEFKVSAQFLE   149
kif1a       YTMMGKQEK---------DQQGIIPQLCEDLFSRINDTT-------NDNMSYSVEVSYME   148
kif5b       HTMEGKLHDP--------EGMGIIPRIVQDIFNYIYSM--------DENLEFHIKVSYFE   136
kif3a       FTMEGVRAIP--------ELRGIIPNSFAHIFGHIAKA--------EGDTRFLVRVSYLE   151
kif11       FTMEGERSPNEEYTWEEDPLAGIIPRTLHQIFEKLT----------DNGTEFSVKVSLLE   162
            .**                  ****.   .:*  :             .  : : .. :*

kif21b      LYNEEILDLFDSTRDPDTRHRRSNIKIHEDANGGIYTTGVTSRLIHSQEELIQCLKQGAL   209
kif1a       IYCERVRDLLNPKNKGN-----LRV--REHPLLGPYVEDLSKLAVTSYNDIQDLMDSGNK   201
kif5b       IYLDKIRDLLDVSKTN------LSV--HEDKNRVPYVKGCTERFVCSPDEVMDTIDEGKS   188
kif3a       IYNEEVRDLLGKDQT--QR---LEV--KERPDVGVYIKDLSAYVVNNADDMDRIMTLGHK   204
kif11       IYNEELFDLLNPSSDVSER---LQMFDDPRNKRGVIIKGLEEITVHNKDEVYQILEKGAA   219
            :* :.: **:.             :             .     : . :::   :  *

kif21b      SRTTASTQMNVQSSRSHAIFTIHLCQMRMCTQPDLVNEAVTGLPDGTPPSSEYETLTAKF   269
kif1a       ARTVAATNMNETSSRSHAVFNIIFTQKRHDAETNI-----------------TTEKVSKI   244
kif5b       NRHVAVTNMNEHSSRSHSIFLINVKQENTQT---------------------EQKLSGKL   227
kif3a       NRSVGATNMNEHSSRSHAIFTITIECSEKGIDGN------------------MHVRMGKL   246
kif11       KRTTAATLMNAYSSRSHSVFSVTIHMKETTIDGE------------------ELVKIGKL   261
             * .. * **  *****::* : .   .                             .*:

kif21b      HFVDLAGSERLKRTGATGERAKEGISINCGLLALGNVISALGDQ---------SKKVVHV   320
kif1a       SLVDLAGSERADSTGAKGTRLKEGANINKSLTTLGKVISALAEMDSGPNKNKKKKTDFI   304
kif5b       YLVDLAGSEKVSKTGAEGAVLDEAKNINKSLSALGNVISALAEG------------STYV   275
kif3a       HLVDLAGSERQAKTGATGQRLKEATKINLSLSTLGNVISALVDG-----------KSTHV   295
kif11       NLVDLAGSENIGRSGAVDKRAREAGNINQSLLTLGRVITALVE------------RTPHV   309
             :*******.   :**  .    *.  .** .* :**.**:** :               .:

kif21b      PYRDSKLTRLLQDSLGGNSQTIMIACVSPSDRDFMETLNTLKYANRARNIKNKVVVNQDK   380
kif1a       PYRDSVLTWLLRENLGGNSRTAMVAALSPADINYDETLSTLRYADRAKQIRCNAVINED-   363
kif5b       PYRDSKMTRILQDSLGGNCRTTIVICCSPSSYNESETKSTLLFGQRAKTIKNTVCVNVEL   335
kif3a       PYRNSKLTRLLQDSLGGNSKTMMCANIGPADYNYDETISTLRYANRAKNIKNKARINED-   354
kif11       PYRESKLTRILQDSLGGRTRTSIIATISPASLNLEETLSTLEYAHRAKNILNKPEVNQKL   369
            ***:* :* :*::.***. :* :    .*:. :  ** .** :..**: *  .  :* .
```

Supplemental Figure S1. Multiple sequence alignment of selected kinesins generated using Clustal Omega [1]. KIF1A residues R216, R254, and R307 are highlighted in red.

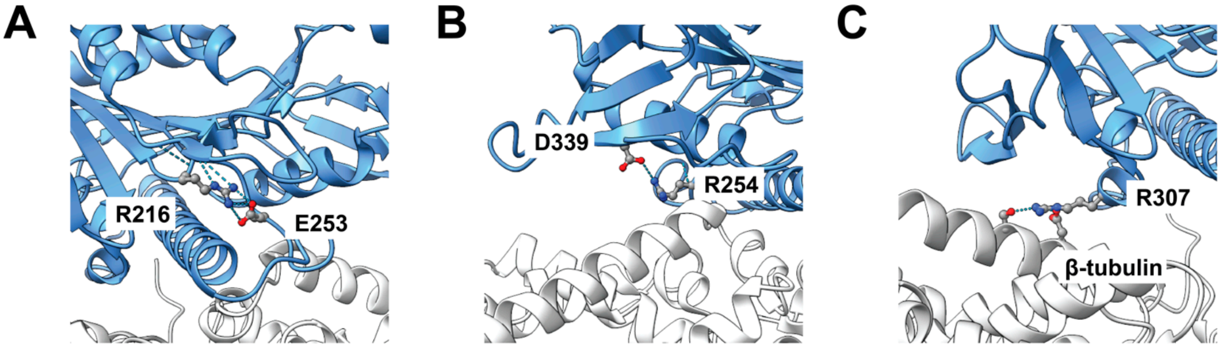

Supplemental Figure S2. Local interactions of KIF1A residues R216, R254, and R307 in the ADP state when bound to microtubules (PDB 8UTR) [2]. **(A)** R216 interacts with E253. **(B)** R254 interacts with D339. **(C)** R307 interacts with S413 and D417 in β-tubulin.

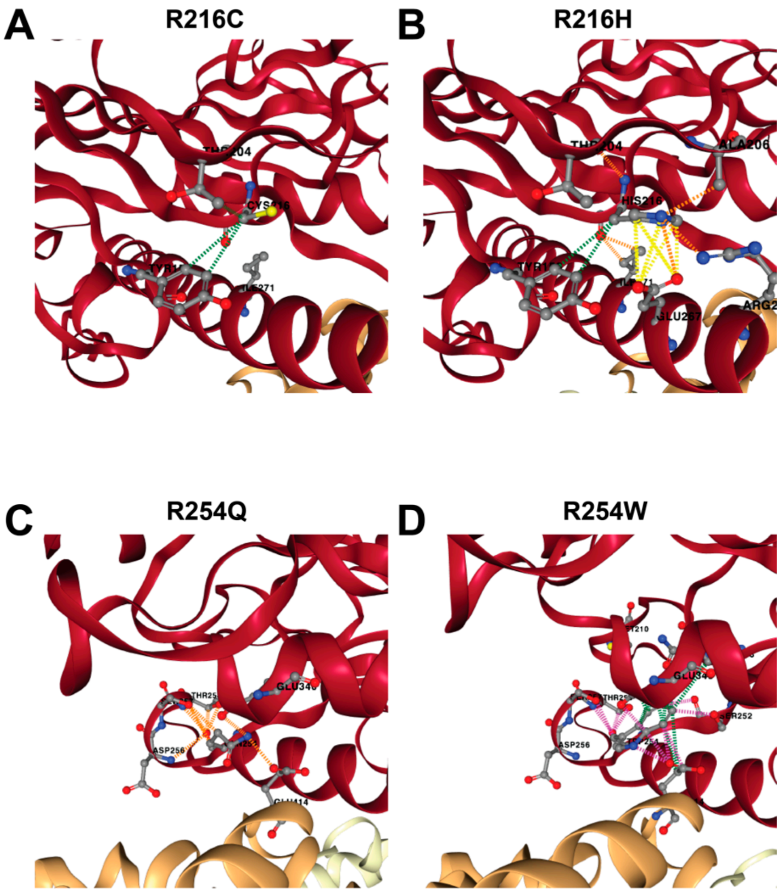

Supplemental Figure S3. Structural prediction of KIF1A mutants using graph-based deep learning.

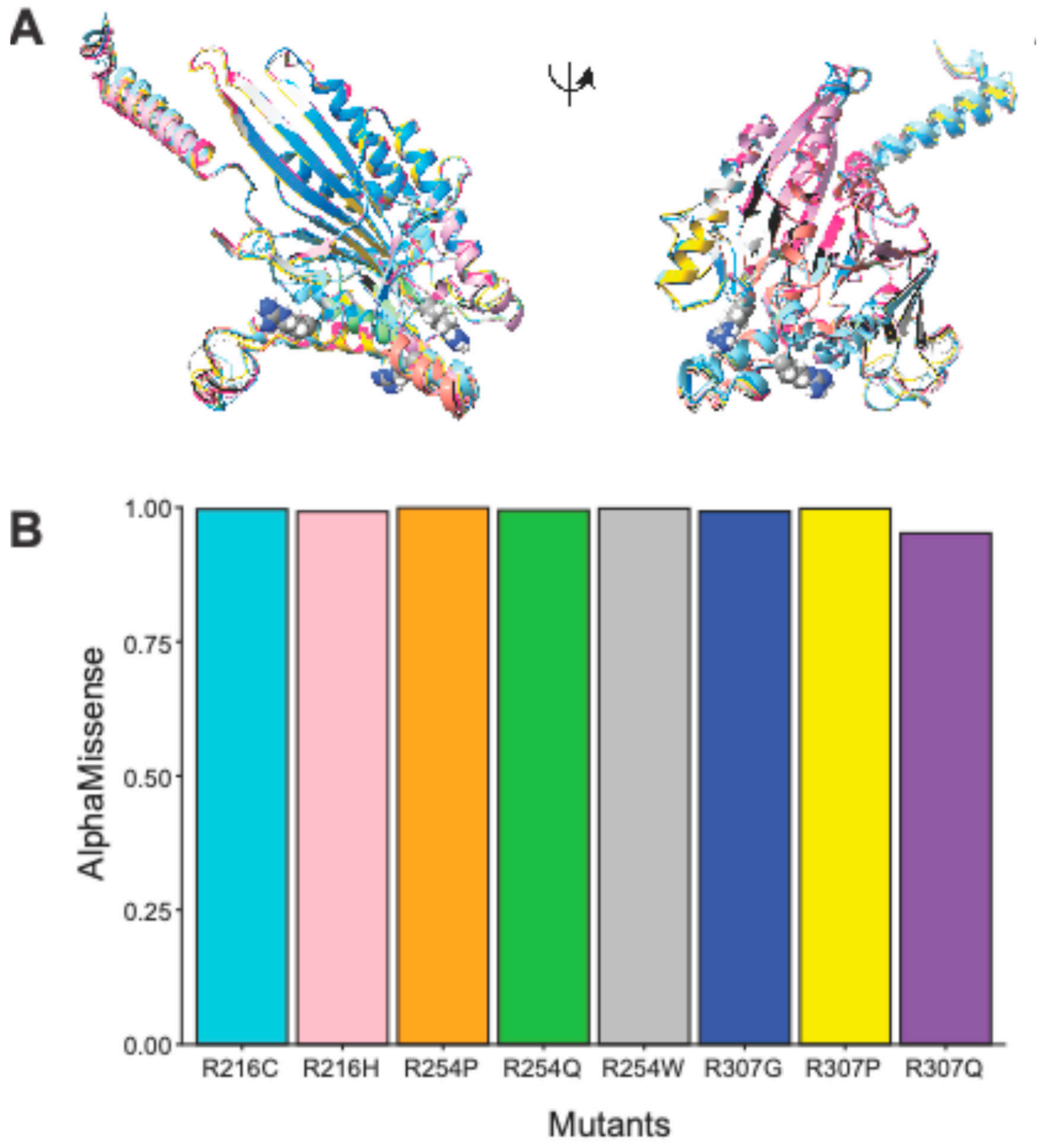

Supplemental Figure S4. Structural and Functional Predictions for Disease-Associated KIF1A Mutations. **(A)** Overlay of AlphaFold2-predicted structures of wild-type and mutant KIF1A motor domains. Residues R216, R254, and R307 are shown in space-filling representation. Color coding: black, wild-type; cyan, R216C; pink, R216H; green, R254Q; orange, R254P; gray, R254W; purple, R307Q; yellow, R307P; and blue, R307G. **(B)** AlphaMissense score for the KIF1A mutations analyzed in this study. Higher scores indicate a greater predicted impact on protein function.

[1] F. Madeira *et al.*, "The EMBL-EBI Job Dispatcher sequence analysis tools framework in 2024," *Nucleic Acids Res,* vol. 52, no. W1, pp. W521-W525, Jul 5 2024, doi: 10.1093/nar/gkae241.
[2] M. Benoit, L. Rao, A. B. Asenjo, A. Gennerich, and H. Sosa, "Cryo-EM unveils kinesin KIF1A's processivity mechanism and the impact of its pathogenic variant P305L," *Nat Commun,* vol. 15, no. 1, p. 5530, Jul 2 2024, doi: 10.1038/s41467-024-48720-4.
